# The burden of neurological impairments and disability in older children measured in disability-adjusted life-years in rural Kenya

**DOI:** 10.1371/journal.pgph.0000151

**Published:** 2022-02-10

**Authors:** Jonathan A. Abuga, Symon M. Kariuki, Amina Abubakar, Samson M. Kinyanjui, Michael Boele van Hensbroek, Charles R. Newton

**Affiliations:** 1 Department of Clinical Research (Neurosciences), KEMRI Wellcome Trust Research Programme, Kilifi, Kenya; 2 Amsterdam Centre for Global Child Health, Emma Children’s Hospital, Academic Medical Centre, University of Amsterdam, Amsterdam, The Netherlands; 3 Department of Public Health, Pwani University, Kilifi, Kenya; 4 Department of Psychiatry, University of Oxford, Oxford, United Kingdom; 5 Institute for Human Development, The Aga Khan University, Nairobi, Kenya; 6 Nuffield Department of Medicine, University of Oxford, Oxford, United Kingdom; University of the Witwatersrand, SOUTH AFRICA

## Abstract

Neurological impairment (NI) and disability are common in sub-Saharan Africa (SSA), but the overall burden in terms of morbidity and mortality in older children remains unknown. We estimated the burden of NI in disability-adjusted life years (DALYs), years of life lost to premature mortality (YLLs), and years lived with disability (YLDs) for older children in a defined rural setting in Kenya. We used empirical and literature estimates to model the overall burden for children aged 5–14 years in five domains: epilepsy (lifetime and active) and moderate/severe cognitive, hearing, motor, and visual impairments. We obtained internally consistent estimates of prevalence, mortality, and transitional hazards using DisMod II software. Disability weights and life expectancy estimates were based on the global burden of disease (GBD) studies. We used the most plausible parameters to calculate YLLs, YLDs, and DALYs and their bootstrapped 95% uncertainty intervals (95%UI) for the defined area. NI in the five domains resulted in a total of 4587 (95%UI 4459–4715) absolute DALYs or 53 (95%UI 39–67) DALYs per 1000 children aged 5–14 years, of which 83% were YLLs and 17% YLDs. Girls had significantly more YLLs and DALYs than boys (p-values <0.001, respectively). Besides being the leading cause of fatal and non-fatal outcomes, epilepsy accounted for the greatest proportion of the total burden for a single domain (20 DALYs per 1000, 95%UI 11–26, or 38.5% of the total DALYs). Visual impairment accounted for the least proportion of the total burden (6 per 1000, 95%UI 1–17, or 12.1%). Children with NI and disability bear a significantly high burden of fatal and non-fatal outcomes. The burden is highest among girls and those with childhood-onset epilepsy. We recommend active identification, treatment, and rehabilitative support for the affected children to prevent premature mortality and improve their quality of life.

## Introduction

The prevalence of neurological impairment (NI) and disability range from 8–180 per 1,000 children in low- and middle-income countries (LMICs) [1]. This high prevalence may be explained by a high incidence of risk factors such as adverse perinatal and neonatal events [2] and malnutrition [3]. Besides, the epidemiology of NI may be changing with a decline in under-five mortality [4], whereby improved child survival due to better control of infections [5], has increased the burden of neurodisability among older children [6]. Children with NI not only have an increased risk for other medical and psychiatric comorbidities [7] but also have a higher risk of premature mortality compared with their peers in the general population [8].

The Global Burden of Disease (GBD) 2017 study highlights that sub-Saharan Africa (SSA) and South Asia bear the highest burden of disability from epilepsy, developmental intellectual disability, hearing loss, and visual impairment for the ages below 20 years, though this was based on limited morbidity data [9]. The number of older children with NI and disability has significantly increased over the past three decades in LMICs [10]. However, these reports might underestimate the overall burden of NI since there was no data for motor impairments. Also, data on the burden of premature mortality was unavailable due to a paucity of epidemiological evidence. The burden of NI in the paediatric population has received little attention in SSA, where morbidity and mortality data are increasingly becoming available [11].

Previous GBD estimates are based on extrapolated epidemiological parameters. These estimates may not accurately represent the true burden and geographical variations at the regional level in the absence of empirical data. A burden of NI study using local data might better inform resource allocation and the development of cost-effective preventive, curative, and rehabilitative interventions for the affected children and their families. We estimated the overall burden caused by common domains of childhood-onset NI in disability-adjusted-life-years (DALYs), years lived with disability (YLDs), and years of life lost (YLLs) for children aged 5–14 years in a demarcated rural setting in Kenya.

## Materials and methods

### Study design and setting

This modelling study utilised data from epidemiological studies conducted between 2001 and 2020 in the Kilifi Health and Demographic Surveillance System (KHDSS). The KHDSS, a designated rural area of about 900 km^2^ located along the coastal region of Kenya (https://kemri-wellcome.org/programme/health-research-linked-to-a-demographic-surveillance-system), provided the sampling frame for the previous epidemiological studies (providing epidemiological data for this analysis) to accurately estimate the incidence, prevalence and premature mortality associated with NI. Since 2000, the KHDSS is re-enumerated two to three times a year by a community census to update vital statistics such as births, deaths, and migration status [12].

### Sources of data

Epidemiological studies in the KHDSS have defined NI as the presence of epilepsy, or deficits in cognitive, vision, hearing, and motor functions among older children following a seminal community survey conducted in 2001 [13]. The assessment and determination of the prevalence and risk factors for childhood-onset NI and disability has previously been described [13, 14]. In summary, children with NI, from these previous community surveys were identified in a two-stage design [15] involving screening a random sample using the Ten-Question Questionnaire (TQQ) in the community [16, 17], followed by comprehensive clinical and neuropsychological assessments at a research hospital [13, 14]. Except for epilepsy where the sensitivity is 100% [18], the TQQ has low sensitivity for mild cognitive, hearing and vision impairments, which are quite difficult to detect in community surveys [17]. Consequently, this modelling study focused on the burden of moderate/severe impairments in these domains for which empirical data was entirely available.

This modelling study utilised prevalence data for children aged 6–9 years from two large community surveys; the first study was conducted in 2001 [13] and the second in 2015 [14]. Prevalence data from both surveys were eligible for modelling the overall burden of non-fatal outcomes (YLDs). However, the input data considered in the actual modelling had to be internally consistent considering known relationships between incidence, prevalence, duration, and mortality [19]. No studies have directly estimated the incidence of cognitive, hearing, vision, and motor impairments in children in the KHDSS since the precise onset of these conditions is difficult to determine, and they are not easily detected in cohort studies. Hence the incidence was calculated from the prevalence, spontaneous remission (assumed to be <1%) and mortality data.

Mortality estimates for the general population were obtained directly from the KHDSS database, which is a prospective routine surveillance system of vital statistics in this defined population [12]. To determine premature mortality among those with NI, we followed up a prospective cohort study of children identified with NI in 2001 [13] until 2018 with a mortality rate of 309.8 per 100 000 person-years of observation (95% CI 126.7–492.9) and a standardised mortality ratio (SMR) of 3.15 (95% confidence interval [CI] 1.66–5.49) reported [20]. A SMR of 6.5 was reported in a similar cohort study that actively followed patients with active convulsive epilepsy in the same study area [21]. We used these surveillance and empirical data to construct age and sex population distribution and the number of expected deaths among children with and without NI for the ages 5–14 years. We have provided additional details of the primary epidemiological studies contributing to this modelling study in S1 Table.

### Disease modelling (input and output parameters)

**Fig 1:**
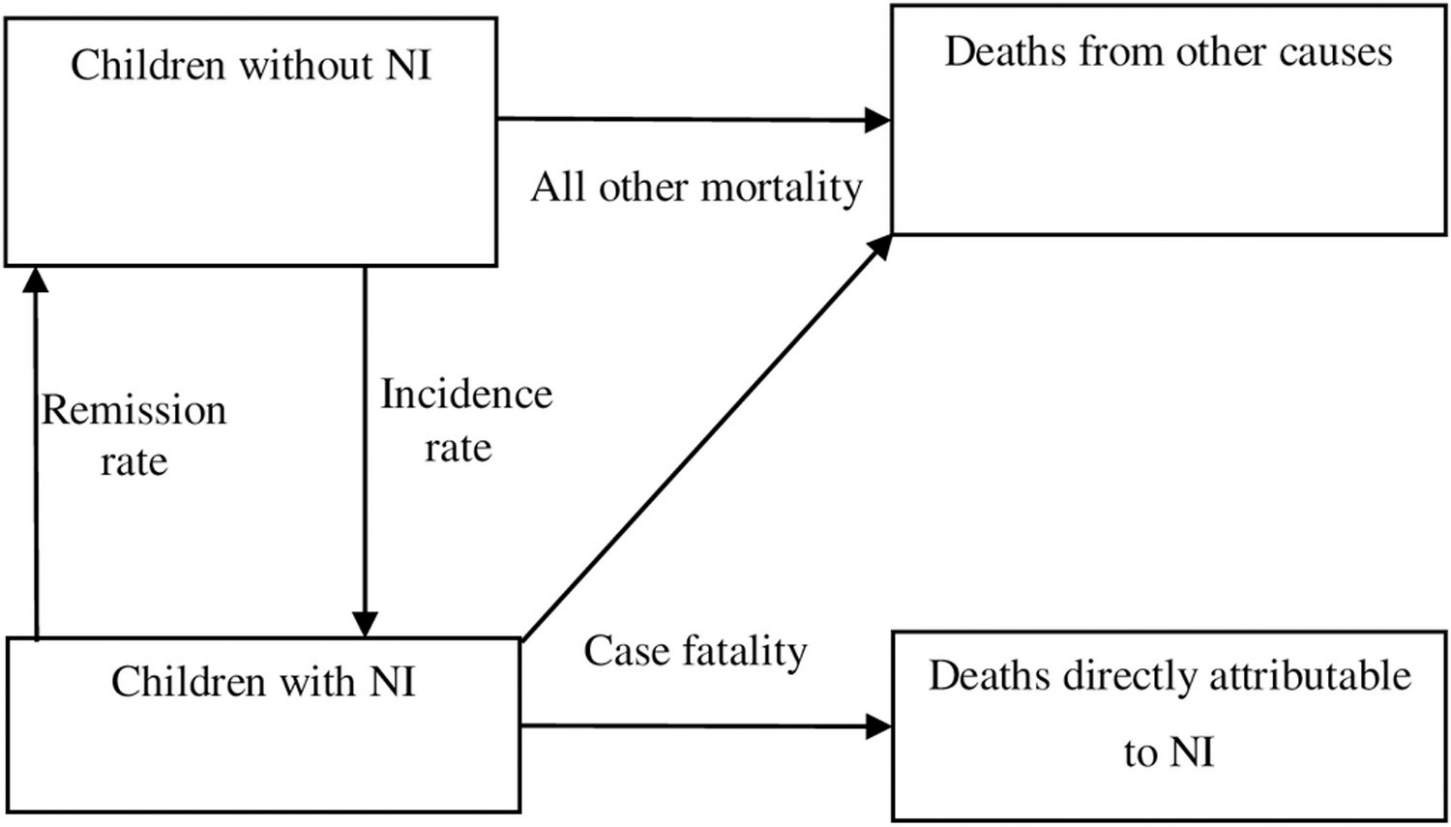
The conceptual model for neurological impairment in DisMod II illustrating three health states and the transitional hazards—incidence, remission, case fatality, and all other mortality.

Internally consistent output parameters were obtained using disease modelling software DisMod II (https://www.who.int/healthinfo/global_burden_disease/tools_software/en/), a model-based approach that utilises known relationships between parameters in the causal structure of a disease process [19]. DisMod II uses a set of differential equations to provide internally consistent estimates of prevalence, mortality, and transitional hazards—incidence, remission, case fatality, and all-cause mortality. The conceptual disease model used for modelling NI in each of the five domains is presented in (Fig 1).

Disease modelling using DisMod II requires at least three input parameters. In this analysis, the input parameters for epilepsy were based on studies conducted in the KHDSS on incidence [22], prevalence [13], and mortality [20, 21]. The other four NI domains are usually life-long conditions that will rarely spontaneously remit. Therefore, we set the remission rate to zero percent for these conditions and used empirical data of prevalence and mortality as the other two additional input parameters. All input and output data for each domain of NI, categorised by age and sex, were individually discussed, and verified by neurodevelopmental experts and neuro-epidemiologists, all of whom are co-authors of this paper, for biological plausibility and comparability with similar studies (S2–S6 Tables).

### Other methodological considerations

Disability weights (DW) are used to convert time lived with a disability to be comparable with time lost to premature mortality [23]. For this analysis, we used the most recent GBD values [24] weighted by the number of children in each severity category. The weighted DW was 0.064 for cognitive impairment, 0.039 for hearing impairment, 0.047 for vision impairment, and 0.171 for motor impairment. We used the values 0.319 and 0.420 for treated epilepsy with recent seizures and untreated epilepsy, respectively, assuming that the overall treatment gap in Kilifi was 62.4% (95%CI 58.1–66.6) [25]. The age of onset of NI in children is usually within the first year of life consequent to adverse pregnancy or birth events and neonatal infections. For this analysis, the age of onset of NI was assumed to be 30 days. We determined premature mortality based on the global standard reference life table used by the 2010 GBD study, which has a life expectancy at birth of 86 years for both males and females [26]. Due to the controversies concerning age-weighting and time-discounting, and to allow for consistency with the GBD studies, we calculated DALYs, YLDs, and YLLs based on uniform age-weighting and a zero percent discounting.

### Ethics statement

This modelling study did not require additional consent since we did not collect new data from the participants. In the original studies, written consent was sought from the parents/guardians of the children who participated in the primary epidemiological studies contributing to this modelling study. The data was stripped on identifying features.

### Statistical analysis

Statistical analysis and computing were done in R statistical programming environment version 3.6.1 [27] using the package ‘DALY’ [28] for stochastic calculation of DALYs, YLDs, and YLLs, and their bootstrapped 95% uncertainty intervals (95%UI). The estimation of 95%UI was based on Monte Carlo simulations with a minimum of 1,000 iterations. All input parameters were set to the beta-Pert probability distribution and output parameters consistently adjusted with the WHO age categories of 5–14 years to allow for comparison across geographical locations, diseases/conditions, and over time. We used the z-test statistics to assess the differences between proportions across the sub-categories included in this analysis because the sample sizes were large and independent.

## Results

A total of 86,360 children aged 5–14 years from the KHDSS, of whom 50.3% (n = 43,454) were males, were involved in this comprehensive analysis. The modelled prevalence for any NI in the five domains was significantly higher in females than males (70.0 per 1000, 95%UI 67.6–72.3 versus 59.9 per 1000, 95%CI 57.7–62.2, p<0.001). The modelled prevalence was highest for cognitive impairment at 27.0 (95%UI 14.8–39.2) per 1000 for males and 35.9 (95%UI 21.1–51.0) per 1000 for females, followed by epilepsy and hearing impairments in that order (Table 1). The modelled estimates of prevalence were lowest for visual impairment at 2.5 per 1000 (95%UI 0.5–4.6). Modelled estimates of incidence, remission, and mortality were highest for epilepsy. A summary of the modelled parameters for the five domains of NI classified by sex for children aged 5–14 years in the KHDSS is presented in Table 1.

**Table 1:** Modelled incidence, prevalence, remission, and mortality parameters by sex and domain of neurological impairment for children aged 5–14 years in Kilifi, Kenya (N = 86,360).

Domain of impairment	Incidence per 100,000 population (95%UI)		Prevalence per 1000 population (95%UI)		Remission rate per 100 children (95%UI)		Standardised mortality ratio (95%UI)	
Sex	Males	Females	Males	Females	Males	Females	Males	Females
Epilepsy	89.06 (31.68–98.01)	88.86 (36.02–97.27)	14.73 (2.57–19.08)	13.83 (2.65–17.05)	18.05 (17.94–25.36)	18.39 (13.11–20.59)	7.64 (2.19–13.03)	7.64 (2.19–13.03)
Moderate/severe cognitive impairment	8.62 (0.01–61.02)	10.22 (0.01–63.46)	26.97(14.81–39.18)	35.94 (21.10–50.97)	0.04 (<0.01–0.11)	0.07 (0.01–0.16)	3.42 (1.35–5.19)	3.52 (1.47–5.28)
Moderate/ severe hearing impairment	0.73 (0.01–51.60)	0.72 (0.01–51.65)	12.00 (6.29–17.74)	15.0 (8.09–21.95)	0.01 (<0.01–0.06)	0.01 (<0.01–0.07)	1.47 (1.01–2.02)	1.46 (1.00–1.94)
Moderate/severe motor impairment	1.66 (<0.01–52.54)	1.21 (0.01–52.14)	5.0 (2.17–7.84)	4.0 (1.56–6.45)	<0.01 (<0.01–0.05)	<0.01 (<0.01–0.05)	3.72 (1.71–6.19)	3.68 (1.33–5.93)
Moderate/severe vision impairment	6.28 (<0.01–54.95)	6.27 (<0.01–56.52)	2.52 (0.45–4.60)	2.52 (0.61–4.43)	<0.01 (<0.01–0.05)	<0.01 (<0.01–0.05)	1.38 (1.00–1.80)	1.37 (1.00–1.77)

NI resulted in a total of 4587.0 (95%UI 4458.9–4715.1) absolute DALYs, of which 83.0% were YLL and 17.0% YLD (Table 2), which was 53.0 DALYs per 1000 (95%UI 38.7–67.2) children aged 5–14 years in the KHDSS (Table 3).

**Table 2:** Absolute estimates of YLL, YLDs, and DALYs (95% Uncertainty Intervals) classified by sex and NI domain for children aged 5–14 years in Kilifi, Kenya.

Domain of impairment	Absolute number of YLL (95%UI)			Absolute number of YLD (95%UI)			Absolute number of DALYs (95%UI)		
Sex	Males	Females	Total	Males	Females	Total	Males	Females	Total
Epilepsy	475.0 (431.8–518.2)	859.0 (800.7–917.3)	1334.0 (1248.2–1388.9)	224.0 (195.4–252.6)	206.0 (178.1–233.9)	430.0 (290.0–572.0)	699.0 (647.3–750.7)	1065.0 (1000.8–1129.2)	1764.0 (1682.6–1845.4)
Cognitive impairment	355.0 (317.0–393.0)	350.0 (313.2–386.8)	705.0 (645.2–748.5)	75.0 (58.5–91.5)	99.0 (80.10–117.89)	174.0 (140.0–208.0)	430.0 (388.8–471.2)	449.0 (407.3–490.7)	879.0 (821.1–936.9)
Hearing impairment	312.0 (276.5–347.5)	302.0 (267.4–336.6)	614.0 (558.2–655.6)	20.0 (11.4–28.6)	25.0 (15.0–35.7)	45.0 (36.0–55.0)	332.0 (296.4–367.6)	327.0 (292.4–361.6)	659.0 (605.9–712.1)
Motor impairment	344.0 (308.6–379.4)	320.0 (283.9–356.1)	664.0 (607.7–704.9)	37.0 (25.5–48.5)	29.0 (18.6–39.4)	66.0 (50.6–81.4)	381.0 (342.9–419.1)	349.0 (311.8–386.2)	730.0 (676.9–783.1)
Vision impairment	281.0 (248.2–313.8)	264.0 (231.7–296.3)	545.0 (496.8–580.6)	5.0 (0.6–9.4)	5.0 (0.6–9.5)	10.0 (7.1–14.5)	286.0 (253.3–318.7)	269.0 (235.9–302.1)	555.0 (509.1–600.9)
Total for the five domains	1767.0 (1683.7–1850.3)	2095.0 (2005.0–2185.0)	3862.0 (3701.3–3933.2)	361.0 (323.9–398.1)	364.0 (325.6–402.4)	725.0 (672.9–777.1)	2128.0 (2036.0–2220.1)	2459.0 (2366.9–2551.1)	4587.0 (4458.9–4715.1)

Abbreviations: YLL, Years of Life Lost; YLD, Years Lived with Disability; DALYs, Disability-Adjusted Life-Years; NI, Neurological Impairment.

**Table 3:** Relative estimates of YLL, YLDs, and DALYs per 1,000 (95% uncertainty intervals) classified by sex and NI domain for children aged 5–14 years in Kilifi, Kenya.

Domain of impairment	YLLs per 1,000 (95%UI)			YLDs per 1,000 (95%UI)			DALYs per 1,000 (95%UI)		
Sex	Males	Females	Total	Males	Females	Total	Males	Females	Total
Lifetime epilepsy	10.9 (4.7–17.3)	20.0 (11.0–29.0)	15.4 (6.1–29.9)	5.2 (4.5–5.8)	4.8 (4.2–5.5)	5.0 (3.0–7.0)	16.1 (8.4–23.6)	24.8 (15.4–34.6)	20.4 (11.0–26.0)
Cognitive impairment	8.2 (2.5–13.5)	8.2 (2.8–13.2)	8.16 (1.2–18.7)	1.7 (1.4–2.1)	2.3 (1.9–2.8)	2.0 (1.5–2.4)	9.9 (4.0–16.0)	10.5 (4.5–17.5)	10.1 (3.0–21.0)
Hearing impairment	7.1 (1.8–12.2)	7.0 (1.9–12.1)	7.1 (1.0–18.0)	0.5 (0.3–0.7)	0.6 (0.3–0.8)	0.5 (0.1–1.0)	7.6 (2.2–13.8)	7.6 (2.2–13.8)	7.6 (1.0–18.9)
Motor impairment	7.9 (1.7–12.3)	7.5 (2.5–13.5)	7.7 (2.6–13.4)	0.9 (<0.1–3.0)	0.7 (<0.1–3.0)	0.8 (<0.1–3.0)	8.8 (3.1–14.9)	8.1 (1.9–14.2)	8.5 (3.1–14.9)
Vision impairment	6.5 (1.7–12.3)	6.2 (0.9–11.11)	6.3 (0.4–17.0)	0.1 (0.01–0.2)	0.1 (0.01–0.2)	0.1 (0.01–0.2)	6.5 (1.8–12.2)	6.3 (1.3–10.7)	6.4 (0.9–16.6)
Total (five domains)	40.7 (28.2–53.8)	48.8 (35.4–62.6)	44.7 (31.3–58.7)	8.0 (2.4–13.6)	8.5 (3.2–14.8)	8.4 (2.6–13.4)	49.0 (35.2–62.8)	57.3 (42.2–71.8)	53.0 (38.7–67.2)

Abbreviations: YLL, Years of Life Lost; YLD, Years Lived with Disability; DALYs, Disability-Adjusted Life-Years; NI, Neurological Impairment.

Boys accounted for 49.0 DALYs per 1000 (95%UI 35.2–62.8) (46.4%), while girls accounted for 57.3 DALYs per 1000 (95%UI 42.2–71.8). Females had a significantly higher burden in terms of YLLs and DALYs (p-values <0.001, respectively), but YLDs were similar for boys and girls (p = 0.78). Epilepsy was the leading cause of the overall NI burden accounting for 38.5% (or 20.4 DALYs per 1000, 95%UI 11.0–26.0) of all DALYs among the children included in this analysis. The burden of epilepsy in DALYs was significantly higher among females than males (p<0.001). Cognitive, motor, hearing, and vision impairments each accounted for 19.2%, 15.9%, 14.4%, and 12.1% of the total DALYs, respectively.

Epilepsy was the leading cause of premature mortality responsible for 34.5% of the total YLLs (or 15.4 YLL per 1000, 95%UI 6.1–29.9). Cognitive, motor, hearing, and vision impairments each accounted for 18.3%, 17.2%, 15.9%, and 14.1% of the total YLL-burden, respectively. Epilepsy was also the leading cause of YLDs accounting for 59.3% of the total YLD-burden. Cognitive, motor, hearing, and vision impairments each accounted for 24.0%, 9.1%, 6.2%, and 1.4% of the total YLDs, respectively. Similarly, YLL and YLD burdens were significantly higher in females than their male counterparts.

## Discussion

Multiple domains of NI cause a substantial burden of disease (53 DALYs per 1000 children aged 5–14 years) in this rural area of Kenya, most of which (83%) are due to YLLs. The overall burden in DALYs differs according to the domain of NI, being highest among children with epilepsy and lowest among those with vision impairments. YLDs were highest among children with epilepsy followed by those with cognitive impairments, conditions with known serious somatic and psychiatric comorbidities. Females had a significantly higher burden in terms of YLLs and DALYs than boys, but YLDs were similar. YLLs were highest among children with epilepsy where mortality is usually high among those with active and untreatable seizures. Inadequate access for treatment and rehabilitative care in LMICs may complicate early-life non-progressive brain damage and associated comorbidities of childhood-onset NI resulting in premature mortality.

These data are important in that they provide region-specific burden estimates that are contextually relevant to inform policy and practice. Earlier [29] and recently updated [10] burden estimates represent the pooled global estimates. All these studies highlight that: (i) the burden may be changing over the years owing to population growth and changes in known risk factors; (ii) epidemiological modelling provides the situation of diseases where empirical studies are unavailable and should be constantly updated; (iii) the increasing contribution of survey data has optimised these epidemiological models; and (iv) there is increased identification, classification and surveillance of NI in LMICs.

The modelled overall prevalence of NI was about 65 per 1000 children aged 5–14 years, which is comparable to the empirically observed estimates of 29–61 per 1,000 for children aged 6–9 years in Kilifi [13]. This data corroborates recent global evidence [10] that prevalence estimates among older children and adolescents are much higher compared to the past WHO estimates of 5.1% for all children [29]. A systematic review [1] reported low estimates because of the over-representation of some NI domains, and this can affect modelled outputs if data from some of these studies were inputted during earlier model development. With an increased contribution of NI data for the GBD studies [11, 30], these models will generate outputs that will be more representative of the contributing settings.

Modelled prevalence was highest for cognitive impairment followed by epilepsy and hearing impairments, respectively; which are consistent with previous empirical studies from this defined area [13]. The lower prevalence of epilepsy compared to cognitive impairment may be explained by the offsets from higher remission rates associated with epilepsy. The high remission rates for epilepsy may be related to better treatment in Kilifi [25], following the set-up of an epilepsy clinic and sensitization initiatives in the community. The low prevalence estimates for visual impairments are probably due to the low sensitivity of the tools to detect mild to moderate visual symptoms [31], and future community surveys for these individual conditions are required.

Absolute and relative estimates for DALYs showed a substantial burden of NI in this defined area on the Kenyan coast. There are no other studies of aggregated DALYs for these domains of NI to compare with since few studies have conducted long-term follow-up for premature mortality, prompting reporting of YLD estimates only [10, 30]. Higher DALYs for epilepsy and motor impairments may be explained by higher DWs than other domains as they are thought to cause more incapacitation and poorer quality of life [24]. Epilepsy alone had 20.4 DALYs per 1000 which is substantial, for children and adolescents, alone, compared to the overall estimate for all age groups of 4.3 per 1000 in the setting [32]. This is consistent with higher prevalence estimates for children than adults [33, 34]. Our estimates were substantially higher than those from a previous epilepsy-burden study conducted in Kilifi [32] because we focused on lifetime epilepsy, which has a higher prevalence than active-convulsive epilepsy. DWs for cognitive, hearing and vision impairments should be reviewed upwards because of the inherent disability and damage they cause to the brain. Higher DALYs/YLLs in females than males could be related to health-seeking behaviours and reporting of severity of health outcomes by the girl’s parents. The overall burden of NI in the studied domains can be reduced through primary prevention, therapeutic management, and rehabilitative services.

The YLD estimates of 8.4 per 1000 are much higher compared with recent global estimates of 2.4–2.7 per 1000 [10] because the latter study used heterogeneous data from fewer and smaller studies conducted in LMICs. The higher burden in YLDs contributed by epilepsy and cognitive impairments (>80%) suggests that these are serious neurological conditions with multiple comorbidities [33]. Lower YLD-burden from hearing and visual domains should be interpreted carefully because there are significantly fewer comorbidity studies for these disorders in LMICs, and that sensorineural conditions have yielded high YLDs in other studies [35]. Lower DWs for cognitive, hearing and vision impairments generally reduced the YLD-estimates in these domains; we recommend longitudinal studies on the presentation and progression of disability associated with the five domains of NI throughout the lifespan to inform the revision of DWs particularly in Africa. There is an urgent need to develop and fund programs for supporting and rehabilitating children with disabilities in their communities to improve the quality of life outcomes.

The relative YLL-burden (44.7/1000) was much higher than YLD-burden (8.4/1000), with the former contributing a substantial 83% of the total DALYs, of which 35% were caused by epilepsy. This is not surprising because mortality is highest among children with untreated epilepsy which might present with comorbid neurological and behavioural disorders [8]. Many studies of NI in the literature did not conduct follow-up for mortality leading to a lack of data for comparison. However, it is not surprising that NIs are associated with a significant burden of YLLs than YLDs. First, YLLs from this study are based on mortality rates from long-term follow-up studies in active KHDSS that provides reliable estimates. Secondly, YLLs considers the years less of the life expectancy of a population, which is greatest for children cohorts such as KHDSS. Finally, YLDs, unlike YLLs, are based on cross-sectional observational of disability associated with a condition, yet YLDs may evolve throughout the lifespan. These relatively higher YLLs than YLDs are similar to those of other conditions like epilepsy from this setting [32]. Lower YLLs for hearing and visual impairments are probably biased by early life deaths that are not captured in prevalence studies in older children.

To the best of our knowledge, this is the first study to investigate the complete epidemiology of NI in DALYs for survival cohorts of children in a defined area in SSA. While the global and regional-level estimates, particularly YLDs, are easily available from the GBD studies, we utilised local empirical data assuming there could be within-region variations that cannot be determined accurately by extrapolating regional-level data. Our data are unique and may provide estimates for rural settings, unlike the GBD estimates that are only generalisable to larger geographical units. The presence of active health and demographic surveillance systems (HDSS) allowed an accurate definition of the population at risk, the measurement of prevalence and incidence, and the determination of mortality rates for children with NI and in the general population for a period of close to two decades.

A major limitation of the GBD modelling approach, which also affects our analyses, is that DALYs are estimated for a single disease or condition. However, most NI and disability in children overlap, for instance, children with epilepsy are more likely to have motor and/or cognitive impairments. It is complicated to estimate the burden caused by comorbidities, for instance, there is no single DW for comorbidities, and it is challenging to determine cause-specific mortality as well. Secondly, it is plausible that YLL estimates in this analysis are overestimated because mortality estimates were based on fewer cohort studies with relatively small sample sizes. Besides, life expectancy in Kilifi, Kenya is much lower compared to the global standard reference life table used by the 2010 GBD study. Also, most deaths in LMICs are not recorded and putative causes of death not identified. These burden estimates represent older children and are not generalisable to other age groups. Results from this study might be generalised to areas similar to Kilifi in terms of human development or the prevalence of infectious and non-infectious risk factors for NI; other epidemiologic studies are required especially from established HDSS sites in LMICs.

## Conclusion

The burden of NI in DALYs, YLLs, and YLDs is high among children older than five years in a defined rural coastal setting in Kenya, with the greatest mortality in girls with NI. Epilepsy accounts for the greatest proportion of both fatal and non-fatal outcomes. These findings might benefit decision-makers in considering potential promotive/preventive, curative, and rehabilitative interventions. This study provides a baseline for future epidemiologic research in LMICs as further scientific evidence is required to improve health and non-health outcomes for children with NI and disabilities as envisaged in the global agenda for children with disabilities, and the Sustainable Development Goals.

## Supporting information

S1 Table Study characteristics of the primary epidemiological studies contributing data to the modeling study. (DOCX)

S2 Table Input and output parameters from DisMod II software for lifetime epilepsy classified by sex for children aged 5–14 years. (DOCX)

S3 Table Input and output parameters from DisMod II software for moderate/severe cognitive impairment classified by sex for children aged 5–14 years. (DOCX)

S4 Table Input and output parameters from DisMod II software for moderate/severe hearing impairment classified by sex for children aged 5–14 years. (DOCX)

S5 Table Input and output parameters from DisMod II software for moderate/severe motor impairment classified by sex for children aged 5–14 years. (DOCX)

S6 Table Input and output parameters from DisMod II software for moderate/severe vision impairment classified by sex for children aged 5–14 years. (DOCX)
