# Supplementary material for: The burden of neurological impairments and disability in older children measured in disability-adjusted life-years in rural Kenya
Source: PLOS Glob Public Health. 2022 Feb 10;2(2):e0000151. doi: 10.1371/journal.pgph.0000151 (PMC7612656; doi:10.1371/journal.pgph.0000151)
Supplement: S1 Table — (DOCX) [file pgph.0000151.s001.docx]

**S1 Table**

| **Reference** | **Parameters** | **Study design** | **Sample size** | **Age categories of the participants** |
| --- | --- | --- | --- | --- |
| Mung’ala et al, 2006 | Prevalence of NI in the five domains | Cross-sectional survey | 10,218 | 6-9 years |
| Abuga et al, 2021 | Prevalence of NI in the five domains | Cross-sectional survey | 11,223 | 6-9 years |
| Abuga et al, 2019 | Premature mortality in the five domains | Prospective cohort study | 10,218 | 6-9 years |
| Ngugi et al, 2014 | Premature mortality in epilepsy | prospective population-based study | 754 | All age groups including children |
| Ngugi et al, 2013 | Incidence of epilepsy | Prospective cohort study | 151,408 | All age groups including children |
| Kariuki et al, 2014 | Age of onset of NI | A multi-site cross-sectional survey | 2,170 | All age groups of whom >51% were children |
| Scott et al, 2012 | Structure of the reference population | A descriptive study of the Kilifi Health and Demographic Surveillance System | 279,158 | All age groups |
