# Supplementary material for: The burden of neurological impairments and disability in older children measured in disability-adjusted life-years in rural Kenya
Source: PLOS Glob Public Health. 2022 Feb 10;2(2):e0000151. doi: 10.1371/journal.pgph.0000151 (PMC7612656; doi:10.1371/journal.pgph.0000151)
Supplement: S2 Table — (DOCX) [file pgph.0000151.s002.docx]

**S2 Table**

|  | **Input parameters** | | | **Output parameters** | | | | | |
| --- | --- | --- | --- | --- | --- | --- | --- | --- | --- |
|  | Incidence per  100, 000 | Prevalence per 1000 | Relative mortality | Incidence rate per 100,000 | Prevalence rate per 1000 | Remission rate (%) | Duration | Mortality rate per 1000 | Relative mortality |
| Males | 95.12 | 21.57 | 6.50 | 89.06  (31.68-98.01) | 14.73  (2.57-19.08) | 18.05  (17.94-25.36) | 2.79  (1.91-3.32) | 0.16  (<0.01-0.17) | 7.64  (2.19-13.03) |
| Females | 95.12 | 20.12 | 6.50 | 88.86  (36.02-97.27) | 13.83  (3.65-17.05) | 18.39  (13.11-20.59) | 2.80  (1.92-3.30) | 0.13  (0.01-0.99) | 7.94  (1.99-14.94) |
