# Supplementary material for: The burden of neurological impairments and disability in older children measured in disability-adjusted life-years in rural Kenya
Source: PLOS Glob Public Health. 2022 Feb 10;2(2):e0000151. doi: 10.1371/journal.pgph.0000151 (PMC7612656; doi:10.1371/journal.pgph.0000151)
Supplement: S3 Table — (DOCX) [file pgph.0000151.s003.docx]

**S3 Table**

|  | **Input parameters** | | | **Output parameters** | | | | | |
| --- | --- | --- | --- | --- | --- | --- | --- | --- | --- |
|  | Prevalence per 1000 | Relative mortality | Remission rate | Incidence rate per 100,000 | Prevalence rate per 1000 | Remission rate (%) | Duration | Mortality rate per 1000 | Relative mortality |
| Males | 27.0 | 3.15 | 0 | 8.62  (0.01- 61.02) | 26.97  (14.81- 39.18) | 0.04  (<0.01- 0.11) | 58.79  (53.91- 61.65) | 0.03  (<0.01- 0.52) | 3.42  (1.35- 5.19) |
| Females | 36.0 | 3.15 | 0 | 10.22  (0.01-63.46) | 35.94  (21.10-50.97) | 0.07  (0.01-0.16) | 62.36  (56.01-66.87) | 0.03  (<0.01-0.50) | 3.52  (1.47-5.28) |
