# Supplementary material for: The burden of neurological impairments and disability in older children measured in disability-adjusted life-years in rural Kenya
Source: PLOS Glob Public Health. 2022 Feb 10;2(2):e0000151. doi: 10.1371/journal.pgph.0000151 (PMC7612656; doi:10.1371/journal.pgph.0000151)
Supplement: S4 Table — (DOCX) [file pgph.0000151.s004.docx]

**S4 Table**

|  | **Input parameters** | | | **Output parameters** | | | | | |
| --- | --- | --- | --- | --- | --- | --- | --- | --- | --- |
|  | Prevalence per 1000 | Relative mortality | Remission rate | Incidence rate per 100,000 | Prevalence rate per 1000 | Remission rate (%) | Duration | Mortality rate per 1000 | Relative mortality |
| Males | 12.00 | 1.30 | 0 | 0.73  (0.01-51.60) | 12.00  6.29-17.74) | 0.01  (<0.01-0.06) | 43.73  (36.75-48.64) | 0.01  (<0.01-0.51) | 1.47  (1.01-2.02) |
| Females | 15.00 | 1.30 | 0 | 0.72  (0.01-51.65) | 15.0  (8.09-21.95) | 0.01  <0.01-0.07) | 49.24  (41.89-55.28) | 0.01  (<0.01-0.50) | 1.46  (1.00-1.94) |
