# Supplementary material for: The burden of neurological impairments and disability in older children measured in disability-adjusted life-years in rural Kenya
Source: PLOS Glob Public Health. 2022 Feb 10;2(2):e0000151. doi: 10.1371/journal.pgph.0000151 (PMC7612656; doi:10.1371/journal.pgph.0000151)
Supplement: S5 Table — (DOCX) [file pgph.0000151.s005.docx]

**S5 Table**

|  | **Input parameters** | | | **Output parameters** | | | | | |
| --- | --- | --- | --- | --- | --- | --- | --- | --- | --- |
|  | Prevalence per 1000 | Relative mortality | Remission rate | Incidence rate per 100,000 | Prevalence rate per 1000 | Remission rate (%) | Duration | Mortality rate/1000 | Relative mortality |
| Males | 5.00 | 3.15 | 0 | 1.66  (<0.01-52.54) | 5.00  (2.17-7.84) | <0.01  (0.01-0.05) | 39.53  (32.98-44.02) | 0.02  (0.01-0.52) | 3.72  (1.71-6.19) |
| Females | 4.00 | 3.15 | 0 | 1.21  (0.01-52.14) | 4.00  (1.56-6.45) | <0.01  (<0.01-0.05) | 48.09  (40.57-54.08) | 0.01  (<0.01-0.51) | 3.68  (1.33-5.93) |
