# Supplementary material for: The burden of neurological impairments and disability in older children measured in disability-adjusted life-years in rural Kenya
Source: PLOS Glob Public Health. 2022 Feb 10;2(2):e0000151. doi: 10.1371/journal.pgph.0000151 (PMC7612656; doi:10.1371/journal.pgph.0000151)
Supplement: S6 Table — (DOCX) [file pgph.0000151.s006.docx]

**S6 Table**

|  | **Input parameters** | | | **Output parameters** | | | | | |
| --- | --- | --- | --- | --- | --- | --- | --- | --- | --- |
|  | Prevalence | Mortality | Remission | Incidence rate per 100,000 | Prevalence rate per 1000 | Remission % | Duration | Mortality rate/1000 | Relative risk mortality |
| Males | 2.60 | 1.30 | 0 | 6.28  (<0.01-54.95) | 2.52  (0.45-4.60) | <0.01  (<0.01-0.05) | 53.51  (47.64-58.98) | <0.01  (<0.01-0.49) | 1.38  (1.00-1.80) |
| Females | 2.60 | 1.30 | 0 | 6.27  (<0.01-56.52) | 2.52  (0.61-4.43) | <0.01  (<0.01-0.05) | 61.27  (55.75-66.50) | 0.01  (<0.01-0.46) | 1.37  (1.00-1.77) |
