# Supplementary material for: The burden of neurological impairments and disability in older children measured in disability-adjusted life-years in rural Kenya
Source: PLOS Glob Public Health. Author manuscript; Available in PMC 2022 Apr 24. (PMC7612656; doi:10.1371/journal.pgph.0000151)
Supplement: Supplementary file 1 [file EMS143814-supplement-Supporting_information_Legends.pdf]

Supporting information

S1 Table. Study characteristics of the primary epidemiological studies contributing data to the modeling study.
(DOCX)

S2 Table. Input and output parameters from DisMod II software for lifetime epilepsy classified by sex for children aged 5–14 years.
(DOCX)

S3 Table. Input and output parameters from DisMod II software for moderate/severe cognitive impairment classified by sex for children aged 5–14 years.
(DOCX)

S4 Table. Input and output parameters from DisMod II software for moderate/severe hearing impairment classified by sex for children aged 5–14 years.
(DOCX)

S5 Table. Input and output parameters from DisMod II software for moderate/severe motor impairment classified by sex for children aged 5–14 years.
(DOCX)

S6 Table. Input and output parameters from DisMod II software for moderate/severe vision impairment classified by sex for children aged 5–14 years.
(DOCX)
